# Correction: A linear classifier based on entity recognition tools and a statistical approach to method extraction in the protein-protein interaction literature

**DOI:** 10.1186/1471-2105-13-180

**Published:** 2012-07-27

**Authors:** Anália Lourenço, Michael Conover, Andrew Wong, Azadeh Nematzadeh, Fengxia Pan, Hagit Shatkay, Luis M Rocha

**Affiliations:** 1 Institute for Biotechnology and Bioengineering, Centre of Biological Engineering, University of Minho, Braga, Portugal; 2 School of Informatics and Computing, Indiana University, Bloomington, IN, USA; 3 FLAD Computational Biology Collaboratorium, Instituto Gulbenkian de Ciência, Oeiras, Portugal; 4 School of Computing, Queen’s University, Kingston, ON, Canada; 5 Microsoft Corp, Redmond, WA, USA; 6 Dept. of Computer and Information Sciences, University of Delaware, Newark, DE, USA; 7 Center for Bioinformatics and Computational Biology, Delaware Biotechnology Institute, University of Delaware, Newark, DE, USA

## Abstract

Correction to A. Lourenço, M. Conover, A. Wong, A. Nematzadeh, F. Pan, H. Shatkay, and L.M. Rocha."A Linear Classifier Based on Entity Recognition Tools and a Statistical Approach to Method Extraction in the Protein-Protein Interaction Literature". *BMC Bioinformatics* 2011, 12(Suppl 8):S12. doi:http://10.1186/1471-2105-12-S8-S12.

## Correction

While reproducing the experiments that we have previously conducted as part of the Article Classification Task (ACT) of the Biocreative III Challenge (BC3), we discovered two errors in our reported results:

1. When computing the performance of two of our four classifiers (VTT3 and VTT5)on the test data, information from class labels was indirectly utilized. This accidental contamination occurred via the additional named entity recognition (NER) features included in these two affected classifiers. Therefore, the performance we previously reported for these two classifiers on test data is higher than it should be. The problem only applies to the test runs under the two classifiers VTT3 and VTT5. Performance reported on the training data for all classifiers and on the test data for the other classifiers remains correct and was not affected by this issue.

2. The values of the area Under the interpolated Precision and Recall Curve (AUCiP/R) performance measure for the test data were reported lower than their true and correct values. This occurred because the official BC3 evaluation script uses the classifier confidence values only if the appropriate variable is checked, which we did not previously do.

Tables 5, 6, and 7 of the original paper [1], which included the affected results, have now been corrected and are attached below.

**Table 5:** Performance of the submitted classifiers over the test data

**Classifier**	**Features**	** *F* **_**1**_	** *Accuracy* **	** *MCC* **	** *AUCiP/R* **
VTT^0^	SP	.5399	.8097	.456	.5399
VTT^0^	Bigrams	.5243	.8382	.4318	.5117
VTT^1^	SP	.5667	.8213	**.4909**	.5843
VTT^1^	Bigrams	.5575	**.8402**	.472	.5769
VTT^5^	SP	.5502	.8378	.4629	.5654
VTT^5^	Bigrams	.5265	.8300	.4336	.536
VTT^3^	SP	**.5682**	.8265	.4906	**.5879**

Values obtained over the official BC3 gold standard using the F-Score, Accuracy, Matthew’s Correlation Coefficient, and Area Under the interpolated Precision and Recall Curve (computed with the official script, and adding F-Score). The highest value for each measure is shown in boldface.

**Table 6:** Summary statistics and variation of the performance of all runs submitted to ACT on the official BC3 gold standard, including our original and our corrected runs

	** *Accuracy* **	** *F* **_** *1* **_	** *MCC* **	** *AUCiP/R* **
Mean	.7906	.4606	.3857	.5046
Median	.8382	.5399	.46	.5367
St. dv.	.1309	.1696	.1696	.1445
Mean + 95% CI	.8247	.5048	.4299	.5422
St. error	.017	.0221	.0221	.0188

Values obtained using the F-Score, Accuracy, Matthew’s Correlation Coefficient, and Area Under the interpolated Precision and Recall Curve (computed with the official script, adding F-Score).

**Table 7:** Performance of top 20 reported runs for the ACT in BC3

**Team**	**Run**	** *Acc.* **	** *Rank* **	** *F* **_** *1* **_	** *Rank* **	** *MCC* **	** *Rank* **	** *AUCiP/R* **	** *Rank* **	** *RP4* **
*T73*	RUN_2	**.8915**	**1**	**.6132**	**2**	**.55306**	**1**	**.6796**	**2**	**4**
T73	RUN_4	.8888	3	**.6142**	**1**	**.55054**	**2**	**.6798**	**1**	**6**
T73	RUN-1	.8755	16	.6083	3	.53524	3	.6591	3	432
*T73*	RUN_3	.8778	13	.6014	6	.52932	6	.6589	4	1872
*T73*	RUN_5	.8762	15	.6033	5	.53031	5	.6537	5	1875
T90	RUN_3	.8832	9	.5964	8	.52914	7	.6524	6	3024
T65	RUN_2	.8793	12	.5982	7	.52727	11	.6389	7	6468
T100	RUN_2	.8827	10	.5949	10	.52732	10	.6186	12	12000
T89	SRV_8	.8687	19	.6080	4	.53336	4	.4740	44	13376
T90	RUN_4	**.8893**	**2**	.5744	14	.52237	12	.4926	42	14112
T90	RUN_2	.8870	6	.5901	11	.5289	8	.5165	36	19008
T90	RUN-1	.8873	5	.5873	12	.52736	9	.5114	38	20520
T100	RUN-1	.8877	4	.5415	28	.50005	16	.6162	13	23296
T65	RUN_5	.8800	11	.5689	16	.50255	15	.6239	10	26400
T65	RUN-1	.8868	7	.5083	38	.48297	20	.6385	8	42560
T90	RUN_5	.8860	8	.5829	13	.52204	13	.5083	40	54080
T89	RUN_5	.8727	18	.5958	9	.52082	14	.4847	43	97524
T100	RUN_4	.8185	37	.5604	20	.4827	21	.6375	9	139860
T81	VTT3-SP	.8265	33	.5682	17	.49065	19	.5879	17	181203
T81	VTT1-SP	.8213	35	.5667	18	.49089	18	.5843	18	204120

The values obtained on the official BC3 gold standard using the F-Score, Accuracy, Matthew’s Correlation Coefficient, and Area Under the interpolated Precision and Recall Curve (computed with the official script, adding F-Score), as well as their respective ranks. RP4 denotes the rank product of these 4 measures. Boldfaced values represent best and second-best performance values for each measure. Our two best runs are shown at the bottom of the table according to the RP4 measure these runs are ranked 19 and 20 among all runs submitted. Overall, our team (81) ranks 6^th^ among all participating teams.

The above issue does not affect any of the results reported for the *Interaction Method Task* (IMT), nor those reported in tables 1–4 of the ACT.

The corrected results do change some of the conclusions we have drawn in the original paper regarding the ACT, as follows:

1. There is a substantial improvement in the ranking and classification of articles relevant to protein-protein interaction when using the ABNER NER tool [2] over abstracts; this can be seen by comparing the performance of VTT0 (no NER tools) with VTT1 (using ABNER) in Table 5. However, there are only minor gains in performance by applying the additional NER tools NLProt [3] and OSCAR 3 [4] to abstracts; this can be seen by comparing the performance of VTT1 (using ABNER) with VTT3 (using ABNER, NLProt and OSCAR 3) shown in the corrected Tables 5 and 7.

2. Including partially available full-text NER data as reported in the original paper [1], does not lead to classification improvement. Indeed, it hinders the performance of the VTT classifier. As can be seen in the corrected Table 5, VTT3 (without full-text NER features) outperforms VTT5 (with additional full-text NER features extracted with ABNER and the PSI-MI ontology [5]) on all performance measures except accuracy. Therefore, instead of the approximately 3% improvement, which we previously reported, including such full-text data actually leads to a 3-5% drop in performance.

3. Our linear classifier VTT5, which uses abstract and full-text NER features, is not the top classifier and does not outperform the best classifiers submitted to BC3. Our top classifiers are VTT3 and VTT1, which perform at approximately the same level (see Table 5). These two simple, linear classifiers obtain an overall competitive result well above the mean and the 95% confidence interval of the performance of all submissions to BC3 (see corrected Table 5 and 6). However, as can be seen in the corrected Table 7, using the rank product of the four main performance measures, these two classifiers rank 19th and 20th among the 59 runs submitted to BC3,including our own original and post-challenge runs. Based on these results, our team ranks 6th among those participating in the ACT task.

Along with the original submission [1], we provided a URL to demos including all data used in the challenge; the errors reported above were reflected in the demo code. At the same URL, we now provide updated demos, in which the above errors are all corrected (http://cnets.indiana.edu/groups/casci/piare).
